# The 26 S proteasome in *Entamoeba histolytica*: divergence of the substrate binding pockets from host proteasomes

**DOI:** 10.1186/s13104-024-06848-y

**Published:** 2024-08-02

**Authors:** Nidhi Joshi, SK Yasir Hosen, Mohd. Fahad, Anil Raj Narooka, S. Gourinath, Swati Tiwari

**Affiliations:** 1 Present Address: Department of Pharmacology, University of Minnesota, Minneapolis, USA; 2 Present Address: Tata Institute of Fundamental Research, Hyderabad, 500046 India; 3 Present Address: Proteomics Department, Advanced Enzymes Technologies Ltd, Thane, 400604 India; 4 School of Life Sciences, Jawaharlal Nehru University, New Delhi, 110067 India; 5 Molecular Cell Biology Laboratory, School of Biotechnology, Jawaharlal Nehru University, New Delhi, 110067 India

**Keywords:** Amoebiasis, *Entamoeba histolytica*, MG132, 26S proteasomes, Protozoan parasite, Ubiquitin

## Abstract

**Objective:**

Proteasomes are conserved proteases crucial for proteostasis in eukaryotes and are promising drug targets for protozoan parasites. Yet, the proteasomes of *Entamoeba histolytica* remain understudied. The study’s objective was to analyse the differences in the substrate binding pockets of amoeba proteasomes from those of host, and computational modelling of β5 catalytic subunit, with the goal of finding selective inhibitors.

**Results:**

Comparative sequence analysis revealed differences in substrate binding sites of *E. histolytica* proteasomes, especially in the S1 and S3 pockets of the catalytic beta subunits, implying differences in substrate preference and susceptibility to inhibitors from host proteasomes. This was strongly supported by significantly lower sensitivity to MG132 mediated inhibition of amoebic proteasome β5 subunit’s chymotryptic activity compared to human proteasomes, also reflected in lower sensitivity of *E. histolytica* to MG132 for inhibition of proliferation. Computational models of β4 and β5 subunits, and a docked β4-β5 model revealed a binding pocket between β4-β5, similar to that of *Leishmania tarentolae*. Selective inhibitors for visceral leishmaniasis, LXE408 and compound 8, docked well to this pocket. This functional and sequence-based analysis predicts differences between amoebic and host proteasomes that can be utilized to develop rationally designed, selective inhibitors against *E. histolytica.*

**Supplementary Information:**

The online version contains supplementary material available at 10.1186/s13104-024-06848-y.

## Introduction

Regulated protein degradation in eukaryotic cells is carried out by the 26S Proteasome, that recognizes proteins modified by chains of a conserved protein called Ubiquitin (Ub). The 26S Proteasomes belong to an ancient superfamily of barrel-shaped proteases that are ATP-dependent [1, 2]. They are composed of a proteolytic core particle (CP) and a regulatory particle (RP). CP has four heptameric rings of alpha and beta subunits and houses six proteolytic active sites contributed by N-terminal threonine residues of β1, β2, and β5 subunits [3].

Proteasome activity is essential for developmental changes of *Trypanosoma*, *Plasmodium*, *Leishmania, Toxoplasma*, and *Entamoeba* [4–6]. Therefore, efforts have been made in recent times for development of selective inhibitors of parasite proteasomes with promising results [7–9]. *Entamoeba histolytica*, the third leading cause of death due to protozoan parasites [10] has a well-developed, functional ubiquitin-proteasome system (UPS) [11]. Proteasome activity is required for its growth and development [12]. Development of drug resistance [13] and the absence of any new drug against this parasite requires urgent research to develop tools to inhibit parasite proliferation and development to prevent new infections. Selective proteasome inhibitors can fulfil these goals.

The current study was designed towards a comparative sequence analysis of the 26 S proteasome CP beta subunits of *E. histolytica* to identify potential structural and functional differences from human proteasomes. Our analysis revealed a pocket between the β4 and β5 subunits of amoebic proteasomes that shows a higher similarity to a similar pocket in Leishmania than to humans. Proteasome inhibitors that selectively bind to this pocket in *Leishmania* have been developed and are currently in clinical trials [8, 14]. We have evaluated these compounds, LXE408 and Compound 8, along with a known proteasome inhibitor bortezomib, in docking studies with a computational model of β4–β5 subunits of amoeba that we developed. This study highlights the differences between the host and amoebic proteasomes in the catalytic subunits that can be exploited for screening and rationally designing selective inhibitors of *E. histolytica* proteasomes.

## Methods

### Sequence retrieval and multiple sequence alignment

Protein sequences were retrieved from the NCBI database using Uniprot accession numbers (Supplementary Table S1). BLAST tool of NCBI (https://blast.ncbi.nlm.nih.gov/Blast.cgi) was used to search the non-redundant protein sequence (nr) of *Entamoeba histolytica* HM-1: IMSS (taxid: 294,381) using BLOSUM45 substitution matrix. Multiple Sequence Alignments (MSA) were generated using CLUSTAL-O tool [15]. Jalview [16] and MEGA-X [17] were used to analyse motifs and residues.

### Proliferation assay

*E. histolytica* HM1:IMSS and HCT8 cells were cultured in TYI-S33 [18] and RPMI media, respectively. Cells were seeded in 96-well plates, and treated with MG-132 after attachment. Cells were washed after 48 h with phosphate buffered saline followed by MTT assay and absorbance recorded at 570 nm.

### Proteasome activity assay

Crude lysates were analysed for proteasome activity using SUC-LLVY-AMC as fluorescence substrate as per manufacturer’s protocol (Cayman Item no. 10,008,041).

### Homology modelling of β4/β5 subunits of proteasome

The homology models were built using SWISS-MODEL [19]. The template was selected by Global Model Quality Estimate (GMQE) [19] and Quaternary Structure Quality Estimate (QSQE) values [20]. Best quality models were selected based on clash scores, MolProbity score, and query coverage % identity. Energy minimization of the models was done with YASARA [21].

The models were validated using SAVES V6.0 and PROSA. The docking of β4 and β5 subunits was performed with HADDOCK 2.4 using default parameters [22]. The best docked model was selected based on the lowest RMSD from the template.

The AutoDock 4.2 tool [23] was used to dock the inhibitor molecule onto the modelled protein (β4–β5). The LXE408 and bortezomib were docked using the reported residues/surface for their interactions with β4 and β5 subunits of *Leishmania tarentolae* proteasome [24]. The grid dimensions for LXE408 and bortezomib were defined at 78 × 66 × 78 and 72 × 62 × 74, respectively, with a spacing of 0.33Å. All the essential charges were calculated, added and allotted to all the atoms of the modelled protein (β4–β5). For Compound 8 [8], the grid box was fixed at the grid-centre of 142.302 × 117.224 × 96.494 with grid dimension fixed at 62 × 48 × 58 with a spacing of 0.33 Å. Hundred runs were performed and twenty-two clusters out of 100 docking poses were generated by taking a cut-off value of 2.0Å using the default Autodock 4.0 parameters for all dockings, Cluster one was chosen based on the highest docking energy and the most populated pose was selected for further analysis. The best docked structure was selected based on the HADDOCK score.

## Results

### Sequence analysis of *E. histolytica* proteasomes

Comprehensive BLAST search of the *Entamoeba histolytica* HM-1: IMSS (taxid: 294,381) genome identified all the subunits of the 26S proteasome, proteasome chaperones and interacting proteins (PIPs) as expected from the ancient and essential nature of this protease (Supplementary Table S1).

Multiple sequence analysis and domain architecture of the beta subunits showed conservation of domains and features of human counterparts, while displaying some key differences as detailed below.

### Substrate binding pockets

Amoebic beta subunits showed a 31–56% homology to the beta subunits of the humans (Supplementary Table S1). The N-terminal threonine residue of catalytic subunits and residues required for the catalytic activity (Asp17, Lys33) were conserved (Supplementary Fig. S1). The enzyme activity is largely determined by the size, composition, and architecture of the substrate binding pockets formed by the 45th position of β-subunits along with residues at 20, 31, 33, 49, 53 positions [25]. Additionally, residues in S1 pocket at 21 and 45 in β1, and 115, 116 in β5 are replaced in immunoproteasomes resulting in an altered pocket size and activity.

**Fig. 1:**
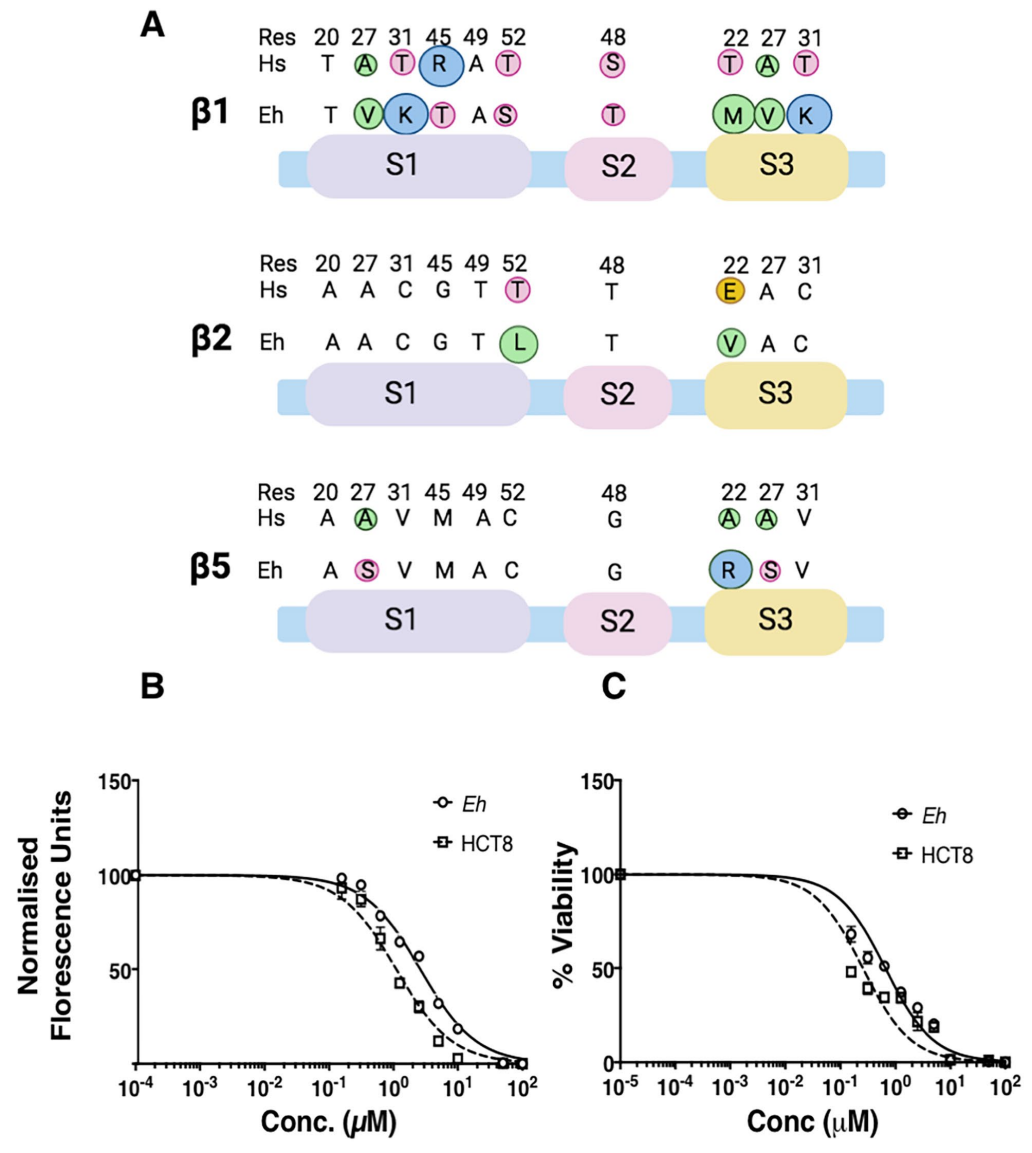
Substrate binding pockets of β1, β2 and β5 subunits of *E. histolytica* and the effect of MG132 on proteasomes activity and cell viability. (**A**) Comparison of human and *E. histolytica* residues present in the S1, S2 and S3 substrate binding pockets of the three catalytic subunits is shown. Size of the circle shows relative size, and residues with similar physiochemical properties are shown in similar colours. Green, hydrophobic; pink, polar; blue, basic; yellow, acidic. **(B)** Normalized and fitted curves for the chymotryptic activity of proteasomes at varying concentrations of MG132 in *E. histolytica* and Hct8 cells. Data are an average of three replicates with bars showing $\pm$SD. **(C)** Normalized and fitted curves of cell viability of *E. histolytica* and Hct8 cells after growing at different concentrations of MG132 for 48 h. Data are an average of three replicates with bars showing $\pm$SD

A comparative of S1, S2 and S3 pockets for the three catalytic subunits of amoeba showed significant differences from host (Fig. 1; Table 1). Amoebic β1 shows substitutions in four out of six residues making up the S1 pocket, two being not conservative replacements. The S2 pocket shows a conservative but slightly smaller residue, while there is a substitution of all three residues in the S3 pocket compared to human β1.

Presence of Gly in S1 pocket of β2 allows a larger pocket that is limited at its lower end by Asp53. In *Entamoeba*, Gly at 45th position is conserved (Fig. 1A) but Asp53, is replaced with Thr at 53rd position (Supp. Fig S1B). The β2 Asp53 forms salt-bridge interactions with basic residues in substrates to give β2 its tryptic-like activity. Threonine cannot participate in salt-bridge formation but can form a hydrogen bond, which has a significantly lower bonding energy compared to a salt-bridge. This may result in a slightly lowered affinity for the basic substrates in amoebic β2. It needs to be experimentally determined if this may result in lower tryptic activity of the amoeba proteasomes compared to human β2 for basic substrates.

Human β5 has Met at 45th position which is conserved in amoeba. But S3 of amoebic β5 is likely to be shallower than human constitutive β5. Additionally, Ala27 is replaced with Ser in amoeba, making it more hydrophilic (Fig. 1A). In immunoproteasomes also, S3 pocket has a Ser instead of Ala.

**Table 1:**
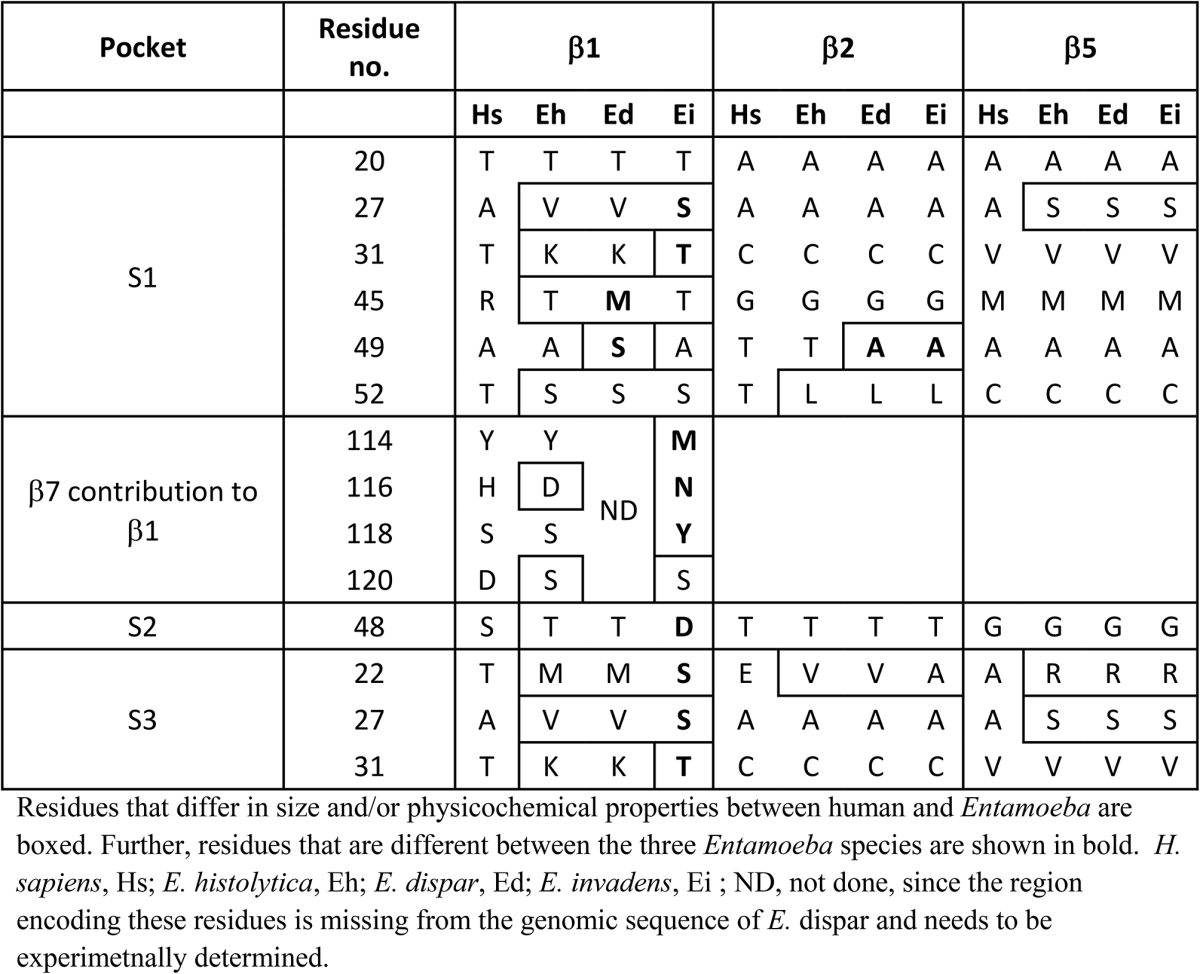
Differences between human and amoebic substrate binding pockets of the catalytic subunits

Moreover, Met45 side chain in the S1 pocket is stabilized by Asn53 in immunoproteasomes which replaces Ser of the constitutive proteasome. In *E. histolytica*, Ala is present at this position, which may increase the distance between the two residues in *E. histolytica* and may have consequences for substrate selection and inhibitor binding. The S3 site of *E. histolytica* has an Arg instead of aliphatic A22 in human β5 (Fig. 1A). This could result in a change in the size and charge of the S3 site. Further, in immunoproteasomes, two residues, Ser115 and Glu116 of human constitutive β5 are substituted with Glu115 and His116. Residues at 115 and 116 are critical for substrate binding and change the substrate cleavage preference of immunoproteasome β5 subunit [26]. *Entamoeba* has Asp and Gly at these positions (Supplementary Fig. S1). Presence of Gly instead of Glu/His will change the polarity and size of the pocket and may allow substrates with larger amino acids to be accommodated by amoebic β5.

This comparative analysis suggests crucial differences between the residues in the substrate binding pockets of the catalytic subunits of human and *Entamoeba*. Interestingly, some differences were also noted in substrate binding pockets between different *Entamoeba* species (Table 1).

### Sensitivity of *E. histolytica* proteasomes to MG132

Significant differences in the substrate binding pockets of amoebic proteasomes prompted us to examine whether amoebic proteasomes have a different sensitivity to peptide-based proteasome inhibitors compared to human proteasomes. A selective, reversible peptide aldehyde,

Carbobenzoxy-L-leucyl-L-leucyl-L-leucinal (MG132) was used to check its effect on the chymotryptic activity of *E. histolytica* proteasomes. The S1 and S3 pockets of β5 subunit of *E. histolytica* is likely to be less hydrophobic than human β5 subunit, and the S3 pocket likely to be smaller due to presence of Arg. The IC50 values for MG132 mediated inhibition of chymotryptic activity of amoebic and human colonic epithelial cell line HCT8 cells were 2.61 and 1.10 µM respectively (Fig. 1B). Thus, *E. histolytica* proteasomes are less sensitive to inhibition by MG132. This was supported by a higher concentration of MG132 that was required to suppress the proliferation of amoebic cells by 50% compared to that needed for HCT8 cells (Fig. 1C). These results strongly support differences in the substrate binding pockets of amoebic proteasomes compared to those of the host cells.

Differential sensitivity of amoebic proteasomes to MG132 supported our hypothesis of structural-functional differences of amoebic proteasomes from host. We used computational modelling to assess other potential differences.

**Fig. 2:**
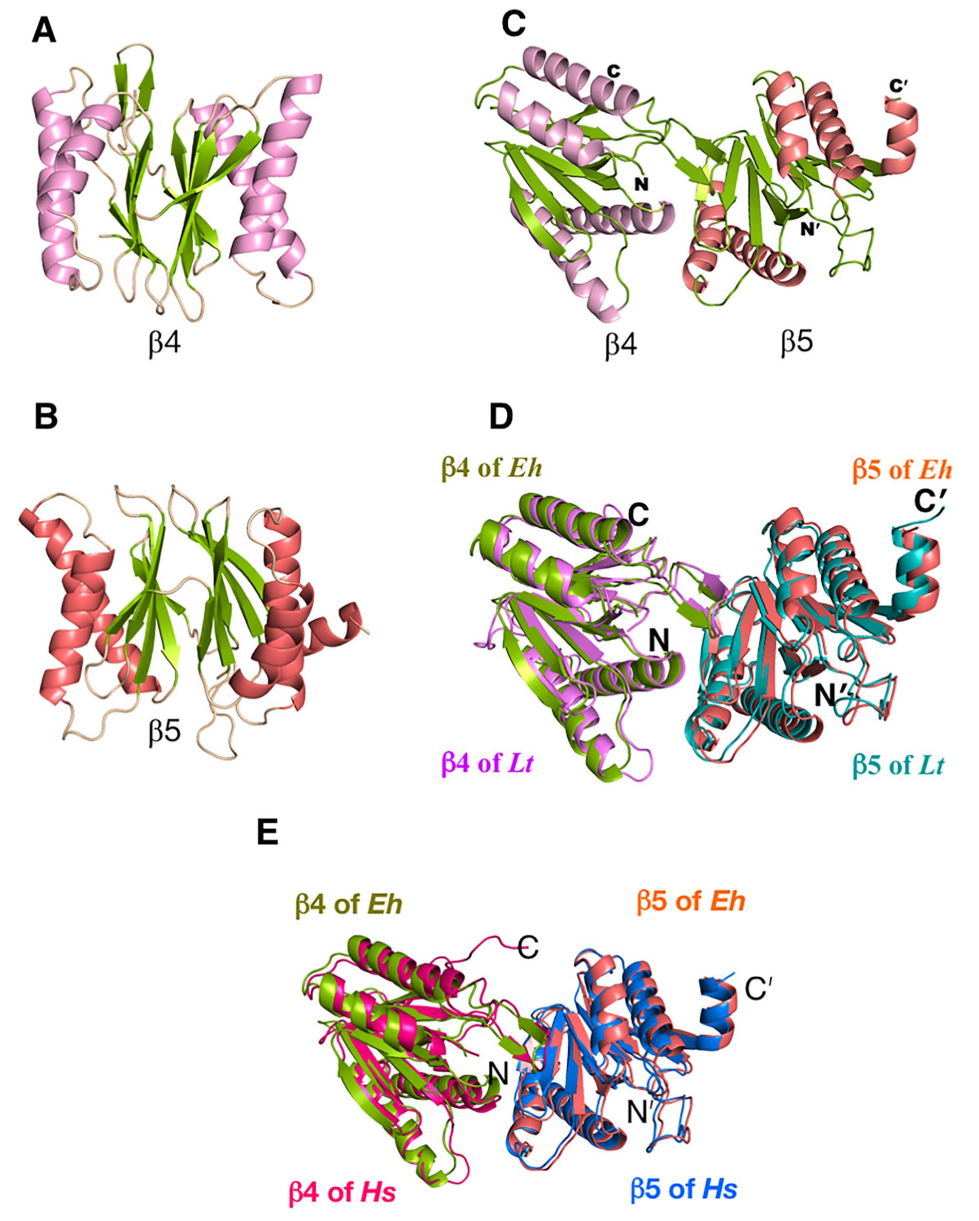
Computational models of β4 and β5 subunits of *E. histolytica*. Representative three-dimensional molecular structures of **(A)** β4, and **(B)** β5 subunits of *E. histolytica.***(C)** Docked model of β4 and β5 of *E. histolytica.***(D)** Overlay of the modelled structure of β4-β5 of *E. histolytica* with *L. tarentolae* and **(E)** human structures. The chains are rendered in the colors indicated for each subunit

### Homology modelling of amoebic β4-β5 subunits

3D-structures of β5 and its adjacent subunit β4, were generated using SWISS-MODEL. *Leishmania tarentolae* proteasome 20S subunit complexed with LXE408 (PDB Id: 6TCZ) and *Leishmania tarentolae* proteasome 20S subunit complexed with bortezomib and LXE408 (PDB Id: 6TD5) structures showed the highest sequence similarity of 34.6% and 53.7% with *Entamoeba histolytica* β4 and β5 subunits, respectively, with about 98% sequence coverage, and the former was chosen as the template to build the models (Fig. 2A, B). Model quality estimates by various parameters collectively established that both models are of good quality and free from steric clashes (Supplementary Fig. S2–S4 and Supplementary Tables S2-S3). *L. tarentolae* shows a pocket between β4 and β5 that has critical differences from the human proteasome (Supplementary Fig. S5) and selective compounds that bind to this pocket in *L*. *tarentolae* and *L. donovani* have been developed that are currently in clinical trial. Since the highest similarity of amoebic proteasome subunits was with the *L. tarentolae* structures, the modelled amoebic structures (β4 and β5) were docked (Fig. 2C) and overlay of the docked modelled structure onto the *L. tarentolae* and human subunits showed Cα RMSD value of ~ 0.96 Å (Fig. 2D, E).

**Fig. 3:**
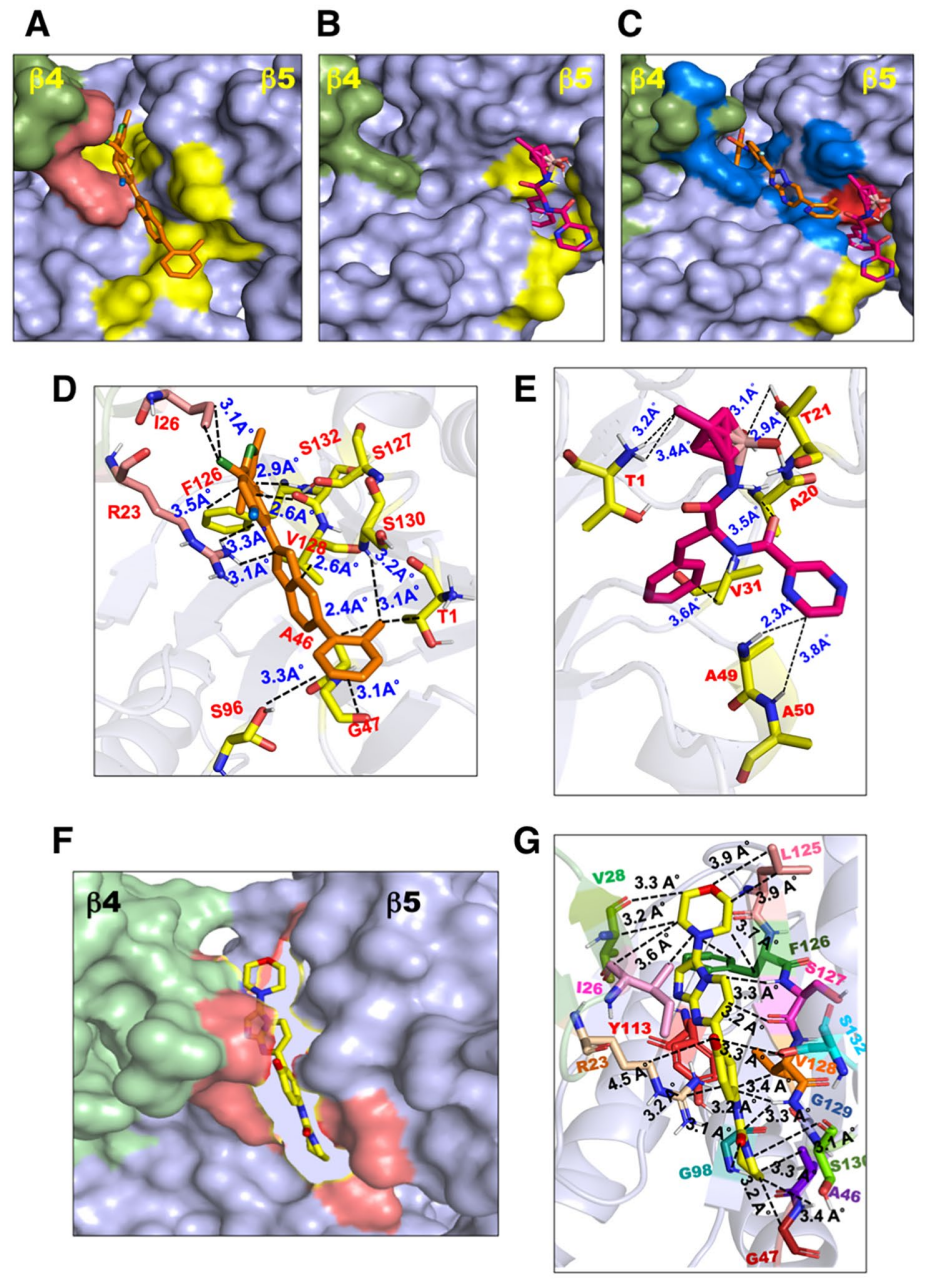
Docking of LXE408, bortezomib and compound 8 onto β4-β5 modeled structure of *Entamoeba histolytica*. The surface structure of the β4-β5 model of *E. histolytica* depicting the binding surface for **(A)** LXE408. The binding pocket has been highlighted in orange and yellow color. **(B)** Binding surface for bortezomib. The binding pocket has been highlighted in yellow color. **(C)** An overlay of LXE408 and Bortezomib inhibitor’s binding surface onto the β4-β5 model of *E. histolytica*. The binding pocket has been highlighted in blue for LXE408 and in red for common LXE408 and bortezomib binding. **(D)** The zoom-in stick representation of the binding pocket of LXE408 and **(E)** bortezomib. All the crucial contacts are depicted by dotted blue lines. **(F)** Surface representation and **(G)** interacting residues of the β4-β5 model of *E. histolytica* with compound 8

The binding of LXE408, bortezomib, and compound 8 to the β4–β5 model using a defined pocket, as well as by blind docking, showed the compounds occupying binding surfaces similar to that observed for the leishmania proteasome (Fig. 3). The estimated binding energies of LXE408 and compound 8 were − 9.5 and − 8.9 kcal mol^–1^, respectively, indicating strong binding. The binding pockets and critical interacting residues for LXE408, bortezomib and compound 8 in *E. histolytica* were conserved compared to those of *Leishmania* (Fig. 3D, E, G). These findings have important implications for development of specific and selective inhibitors against amoebiasis.

## Discussion

Proteasomes have emerged as an attractive target for anti-parasitic drugs as they inhibit the growth and development of many protozoa. Large-scale phenotypic screening for proteasome inhibitors has led to development of several selective inhibitors for trypanosomatids, Leishmania and Plasmodium. *Entamoeba histolytica* proteasome has not received much attention despite amoebiasis being a global disease contributing to 9% of all deaths of children under the age of 5 years [27].

Substitution of a few residues changes the activity of immunoproteasome catalytic subunits. It is likely that the S1 pocket of amoebic β1 has both size and charge differences from the human constitutive and immunoproteasome β1 subunits. Amoebic β5 appears to have a charged, hydrophilic S1 and shallower S3 pocket compared to human β5. Additionally, it may allow substrates with larger amino acids to be accommodated. Our data showing significantly higher IC50 values of amoebic β5 for MG132 is strongly indicative of key differences between amoebic and human proteasomes.

Selective proteasome inhibitors against a number of protozoa have been reported, some, including LXE408 and compound 8, being in clinical trials [8, 26–28]. Since these compounds showed a good docking to our β4/β5 model in the same pocket as in *Leishmania*, it is likely that these, and similar compounds may selectively inhibit amoeba proteasomes. Additionally, differences in the β1 substrate binding pocket can be exploited to develop selective inhibitors.

Our analysis provides a framework to explore the biochemical and structural understanding of amoebic proteasomes that will help in development of selective inhibitors of amoebic proteasomes. Since proteasome activity is essential for developmental changes, these inhibitors will be promising tools to break the infection cycle in amoeba endemic countries.

### Limitations

The conclusions drawn from docked model are limited in that they require confirmation by experimental analysis with compounds similar to LXE408 and compound 8. Determination of proteasome activity, IC50 values, selectivity and specificity analyses are required to validate the results of this study.

### Electronic supplementary material

Below is the link to the electronic supplementary material.

Supplementary Material 1

Supplementary Material 2

## Data Availability

No datasets were generated or analysed during the current study.

## Abbreviations

CP: Core particle
MG132: Carbobenzoxy-L-leucyl-L-leucyl-L-leucinal
GMQE: Global model quality estimate
QSQE: Quaternary structure quality estimate
RP: Regulatory particle
Ub: Ubiquitin
UPS: Ubiquitin-Proteasome system
