# Supplementary material for: The 26 S proteasome in Entamoeba histolytica: divergence of the substrate binding pockets from host proteasomes
Source: BMC Res Notes. 2024 Aug 2;17:216. doi: 10.1186/s13104-024-06848-y (PMC11295364; doi:10.1186/s13104-024-06848-y)
Supplement: Supplementary file 1 — Supplementary Material 1 [file 13104_2024_6848_MOESM1_ESM.docx]

Joshi et al. 26S proteasomes of *E. histolytica*

**Supplementary Tables**

**Supplementary Table S1:** 26S Proteasome, proteasome chaperones and proteasome interacting proteins (PIPs) in *E. histolytica*

|  | **Human** | | ***Entamoeba histolytica* HM1** | | | |
| --- | --- | --- | --- | --- | --- | --- |
| **Systematic Nomenclature** | **HUGO Nomenclature** | **UniProt ID** | **Accession Number (NCBI)/ AmoebaDB Gene ID** | **Query Coverage** | **% Identity** | **E-value** |
| α1 | PSMA6 | P60900 | XP_656396/ EHI_153190 | 98 | 47.11 | 3E-82 |
| α2 | PSMA2 | P25787 | XP_655350/ EHI_052140 | 97 | 58.7 | 1.00E-103 |
| α3 | PSMA4 | P25789 | XP_655561/ EHI_167720 | 91 | 52.08 | 3E-98 |
| α4 | PSMA7 | O14818 | XP_653725/ EHI_163650 | 97 | 55.33 | 1E-97 |
| α5 | PSMA5 | P28066 | XP_650714/ EHI_023020 | 91 | 65.91 | 5E-109 |
| α6 | PSMA1 | P25786 | XP_653086/ EHI_086080 | 93 | 50.14 | 7.00E-93 |
| α7 | PSMA3 | P25788 | XP_649572/ EHI_029210 | 92 | 51.91 | 3E-96 |
| β1 | PSMB6 | P28072 | XP_650977/ EHI_024470 | 83 | 39.2 | 8.00E-53 |
| β2 | PSMB7 | Q99436 | XP_657538/ EHI_148040 | 82 | 44.35 | 4.00E-68 |
| β3 | PSMB3 | P49720 | XP_655858/ EHI_049330 | 99 | 43.35 | 2.00E-64 |
| β4 | PSMB2 | P49721 | XP_651433/ EHI_078710 | 92 | 31.18 | 2.00E-36 |
| β5 | PSMB5 | P28074 | XP_653800/ EHI_011870 | 76 | 56.5 | 1.00E-84 |
| β6 | PSMB1 | P20618 | XP_653346/ EHI_174670 | 85 | 41.26 | 1.00E-54 |
| β7 | PSMB4 | P28070 | XP_652586/ EHI_137970 | 81 | 38.53 | 1.00E-55 |
| Rpn3 | PSMD3 | O43242 | XP_655570/ EHI_179970 | 64 | 44.48 | 1.00E-91 |
| Rpn5 | PSMD12 | O00232 | XP_655243/ EHI_136180 | 79 | 35 | 4.00E-69 |
| Rpn6 | PSMD11 | O00231 | XP_656698/ EHI_182600 | 84 | 37.67 | 5.00E-86 |
| Rpn7 | PSMD6 | Q15008 | XP_651402/ EHI_054230 | 65 | 32.55 | 2.00E-49 |
| Rpn8 | PSMD7 | P51665 | XP_649261/ EHI_030170 | 84 | 43.93 | 1.00E-79 |
| Rpn9 | PSMD13 | Q9UNM6 | XP_653351/ EHI_103850 | 88 | 32.15 | 3.00E-59 |
| Rpn11 | PSMD14 | O00487 | XP_650487/ EHI_164750 | 93 | 61.25 | 2.00E-137 |
| Rpn12 | PSMD8 | P48556 | XP_655395/ EHI_200220 | 31 | 25.23 | 2.00E-08 |
| Rpn1 | PSMD2 | Q13200 | XP_648181/ EHI_198010 | 94 | 34.45 | 1E-179 |
| Rpn2 | PSMD1 | Q99460 | XP_657147/ EHI_049680 | 93 | 39.29 | 2E-156 |
| Rpn10 | PSMD4 | P55036 | XP_653411/ EHI_005870 | 58 | 40.09 | 1.00E-38 |
| Rpn13 | ADRM1 | Q16186 | XP_649366/ EHI_095030 | 13 | 33.93 | 4.00E-05 |
| Rpt1 | PSMC2 | P35998 | XP_001914172/ EHI_080890 | 96 | 69.29 | 0 |
| Rpt2 | PSMC1 | P62191 | XP_653833/ EHI_180350 | 92 | 69.85 | 0 |
| Rpt3 | PSMC4 | P43686 | XP_648721/ EHI_177320 | 91 | 72.77 | 0 |
| Rpt4 | PSMC6 | P62333 | XP_654722/ EHI_194570 | 97 | 63.95 | 0 |
| Rpt5 | PSMC3 | P17980 | XP_657099/ EHI_053020 | 92 | 63.97 | 0 |
| Rpt6 | PSMC5 | P62195 | XP_649078/ EHI_185410 | 98 | 74.37 | 0 |
| P200 | PSME4 | Q14997 | EHI_082520 18.4 0.83 | | | |
|  | PSMF1 | Q92530 | No Significant Similarity | | | |
|  | POMP | Q9Y244 | No Significant Similarity | | | |
|  | PSMG1 | O95456 | No Significant Similarity | | | |
|  | PSMG2 | Q969U7 | XP_651844/ EHI_040290 | | | |
| hUmp1 | PSMG3 | Q9BT73 | No Significant Similarity | | | |
| PAC1 | PSMG4 | Q5JS54 | No Significant Similarity | | | |
| PAC2 | PSMD5 | Q16401 | No Significant Similarity | 45 | 32.5 | 0.000000003 |
| PAC3 | STRN | O43815 | XP_656051/ EHI_118050 | | | |
| PAC4 | PAAF1 | Q9BRP4 | XP_654199/ EHI_140750 | | | |
|  | PSMD9 | O00233 | XP_649580/ EHI_074770 | | | |
| Rpn4 | PSMD10 | O75832 | XP_652130/ EHI_134800 | 41 | 30.33 | 1E-10 |
|  | ECM29 | Q5VYK3 | No Significant Similarity | 51 | 29.11 | 8E-19 |
|  | USP14 | P54578 | XP_651481/ EHI_119600 | 73 | 27.98 | 3E-13 |
|  | UCHL5 | Q9Y5K5 | XP_654194/ EHI_140500 | 86 | 28.37 | 2E-23 |
|  | UBE3C | Q15386 | XP_648644/ EHI_104570 | | | |
|  | UBE3A | Q05086 | XP_653202/ EHI_011530 | 13 | 41.18 | 0.0000003 |
| UCHL5 | PARK2 | O60260 | XP_649010/ EHI_191780 | 93 | 35.65 | 4E-58 |
|  | RAD23A | P54725 | XP_649512/ EHI_001400 | 37 | 31.39 | 5E-53 |
|  | RAD23B | P54727 | XP_649512/ EHI_001400 | 53 | 43.82 | 1E-114 |
|  | UBQLN1 | Q9UMX0 | XP_649202/ EHI_192740 | 27 | 32.53 | 4E-12 |
|  | UBQLN2 | Q9UHD9 | XP_653492/ EHI_022980 | 98 | 26.95 | 9E-23 |

Joshi et al. 26S proteasomes of *E. histolytica*

**Supplementary Table S2:** Comparison of different templates and their parameters used to build the b4-b5 model.

| β**4 Model No.** | **Sequence Similarity** | | **MolProbity** | | **Clash Score** | | **Ramachandran Plot (%)** | | **QMEAN** | | **GMQE** | | **Organism** | |
| --- | --- | --- | --- | --- | --- | --- | --- | --- | --- | --- | --- | --- | --- | --- |
| 01 | 34.57% | | 1.84 | | 4.32 | | 93.55 (favoured) | | -1.03 | | 0.7 | | *Leishmania tarentolae* proteasome 20S subunit complexed with LXE408 | |
| 02 | 34.57% | | 2.26 | | 9.41 | | 94.09 (favoured) | | -2 | | 0.69 | | *Leishmania tarentolae* proteasome 20S subunit complexed with GSK3494245 | |
| 03 | 31.72% | | 1.8 | | 5.45 | | 93.30 (favoured) | | -3.35 | | 0.63 | | *Plasmodium falciparum* 20S proteasome in complex with two PA28 activators | |
| 04 | 31.73 | | 1.83 | | 6.86 | | 92.90 (favoured) | | -4.23 | | 0.63 | | Design of *Plasmodium*-selective proteasome inhibitors | |
| 05 | 34.39 | | 1.84 | | 6.04 | | 93.00 (favoured) | | -0.91 | | 0.7 | | *Leishmania tarentolae* proteasome 20S subunit complexed with LXE408 | |
| 06 | 34.35 | | 2.26 | | 9.4 | | 94.13 (favoured) | | -1.8 | | 0.69 | | *Leishmania tarentolae* with GSK3494245 | |
| 07 | 31.58 | | 1.14 | | 2.33 | | 97.34 (favoured) | | -2.11 | | 0.69 | | Mouse 20S Immunoproteasome | |
| 08 | | 30.5 | | 1.5 | | 2.33 | | 92.02 (favoured) | | -4.79 | | 0.57 | | Human 26S proteasome |
| **β5 Model No.** | | **Sequence Similarity** | | **MolProbity** | | **Clash Score** | | **Ramachandran Plot (%)** | | **QMEAN** | | **GMQE** | | **Organism** |
| 01 | | 53.73 | | 1.65 | | 3.52 | | 91.46 (favoured) | | -1.27 | | 0.57 | | *Leishmania tarentolae* with LXE408 and Bortezomib |
| 02 | | 54 | | 1.63 | | 3.54 | | 91.92 (favoured) | | -1.27 | | 0.57 | | *Leishmania tarentolae* with LXE408 and Bortezomib |
| 03 | | 52.35 | | 1.64 | | 4.16 | | 94.47 (favoured) | | -1.76 | | 0.58 | | *Leishmania tarentolae* with LXE408 |
| 04 | | 53.08 | | 1.61 | | 4.18 | | 94.95 (favoured) | | -1.82 | | 0.57 | | *Leishmania tarentolae* with LXE408 |
| 05 | | 52.34 | | 2.25 | | 7.69 | | 92.46 (favoured) | | -1.11 | | 0.57 | | *Leishmania tarentolae* with GSK3494245 |
| 06 | | 53.08 | | 2.24 | | 7.72 | | 92.93 (favoured) | | -1.03 | | 0.56 | | *Leishmania tarentolae* with GSK3494245 |
| 07 | | 55.67 | | 2.12 | | 6.97 | | 83.58 (favoured) | | -4.28 | | 0.53 | | *Plasmodium falciparum* with PA28 activator |
| 08 | | 46.15 | | 1.87 | | 2.77 | | 90.78 (favoured) | | -1.21 | | 0.59 | | *Saccharomyces cerevisiae* |

Joshi et al. 26S proteasomes of *E. histolytica*

**Supplementary Table S3** Ramachandran plot statistics of β4 and β5 models.

| Ramachandran Plot Statics | β4 | β5 |
| --- | --- | --- |
| Residues in most favoured regions [A,B,L] | 149 (89.8%) | 156 (89.7%) |
| Residues in additional allowed regions [a,b,l,p] | 14 (8.4%) | 16 (9.2%) |
| Residues in generously allowed regions [~a,~b,~l,~p] | 2 (1.2%) | 1 (0.6% ) |
| Residues in disallowed regions | 1 (0.6%) | 1 (0.6% ) |
| Number of non-glycine and non-proline residues | 166 (100.0%) | 174 (100.0% ) |
| Number of end-residues (excl. Gly and Pro) | 2 | 2 |
| Number of glycine residues  (shown as triangles) | 17 | 17 |
| Number of pro-line residues | 3 | 7 |
| Total number of residues | 188 | 200 |
