# Supplementary material for: The 26 S proteasome in Entamoeba histolytica: divergence of the substrate binding pockets from host proteasomes
Source: BMC Res Notes. 2024 Aug 2;17:216. doi: 10.1186/s13104-024-06848-y (PMC11295364; doi:10.1186/s13104-024-06848-y)
Supplement: Supplementary file 2 — Supplementary Material 2 [file 13104_2024_6848_MOESM2_ESM.pptx]

## Slide 1
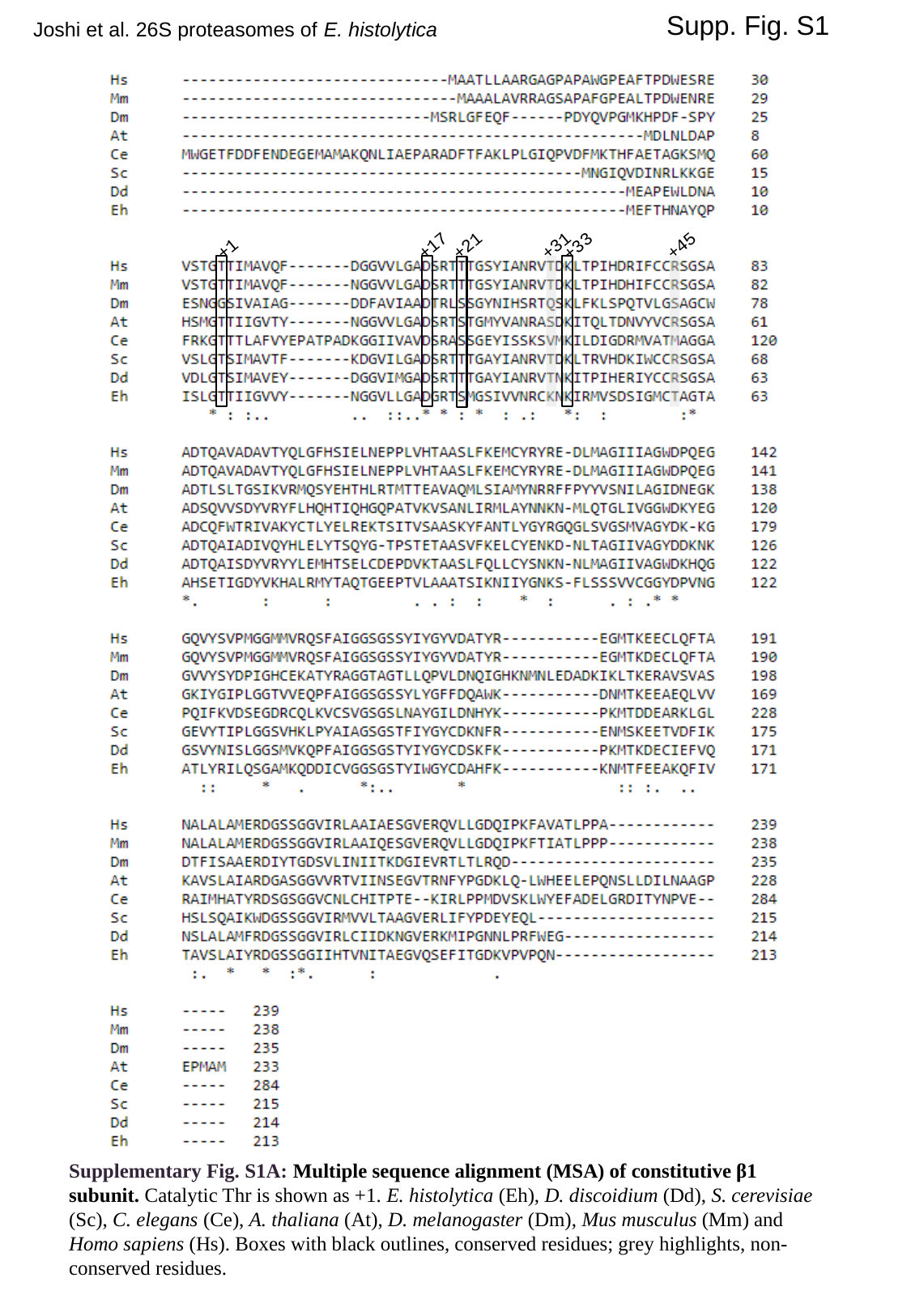

Supp. Fig. S1
Joshi et al. 26S proteasomes of E. histolytica
+17
+21
+31
+45
+33
+1
Supplementary Fig. S1A: Multiple sequence alignment (MSA) of constitutive β1 subunit. Catalytic Thr is shown as +1. E. histolytica (Eh), D. discoidium (Dd), S. cerevisiae (Sc), C. elegans (Ce), A. thaliana (At), D. melanogaster (Dm), Mus musculus (Mm) and Homo sapiens (Hs). Boxes with black outlines, conserved residues; grey highlights, non-conserved residues.

## Slide 2
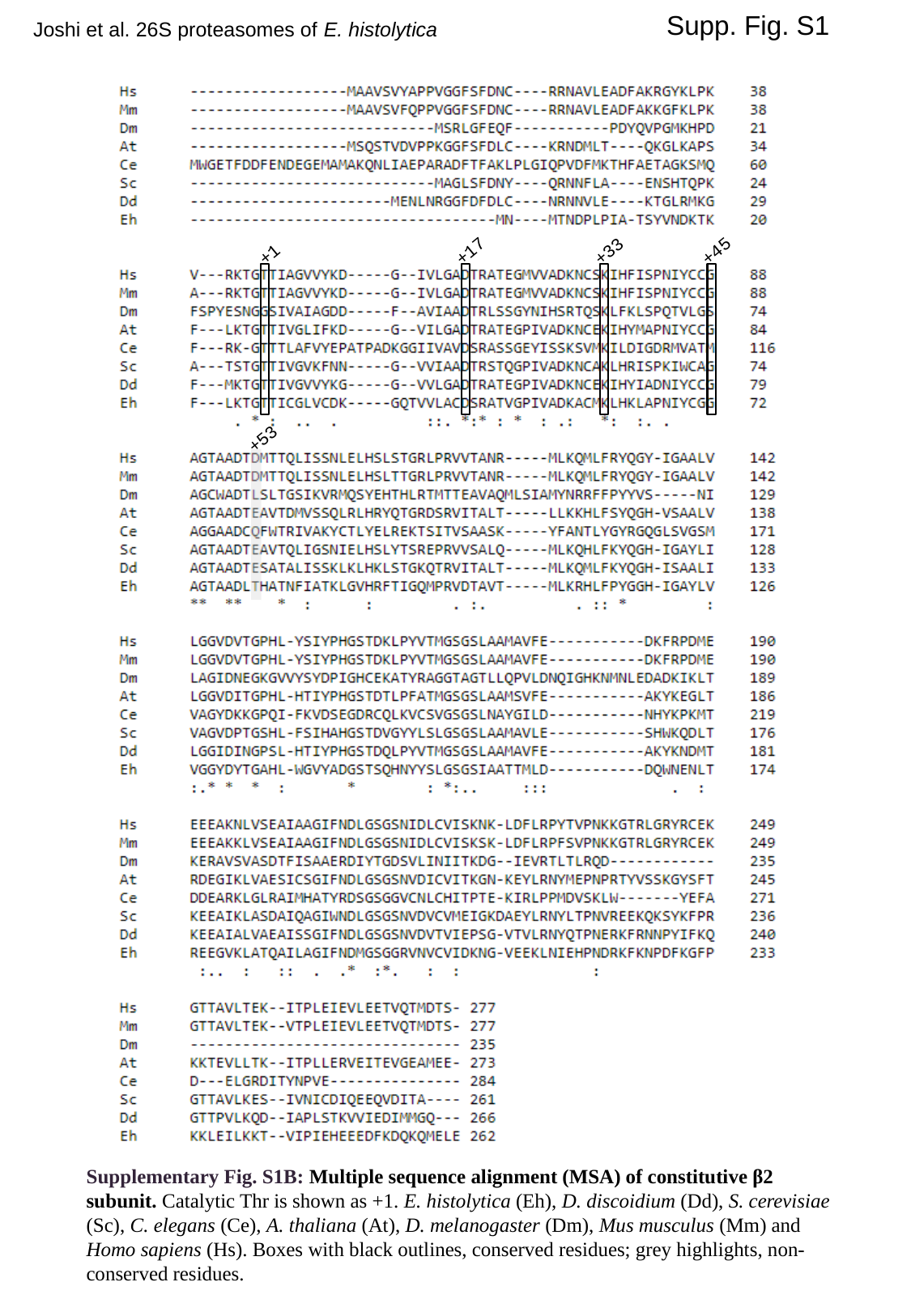

Supp. Fig. S1
Joshi et al. 26S proteasomes of E. histolytica
+17
+33
+45
+1
+53
Supplementary Fig. S1B: Multiple sequence alignment (MSA) of constitutive β2 subunit. Catalytic Thr is shown as +1. E. histolytica (Eh), D. discoidium (Dd), S. cerevisiae (Sc), C. elegans (Ce), A. thaliana (At), D. melanogaster (Dm), Mus musculus (Mm) and Homo sapiens (Hs). Boxes with black outlines, conserved residues; grey highlights, non-conserved residues.

## Slide 3
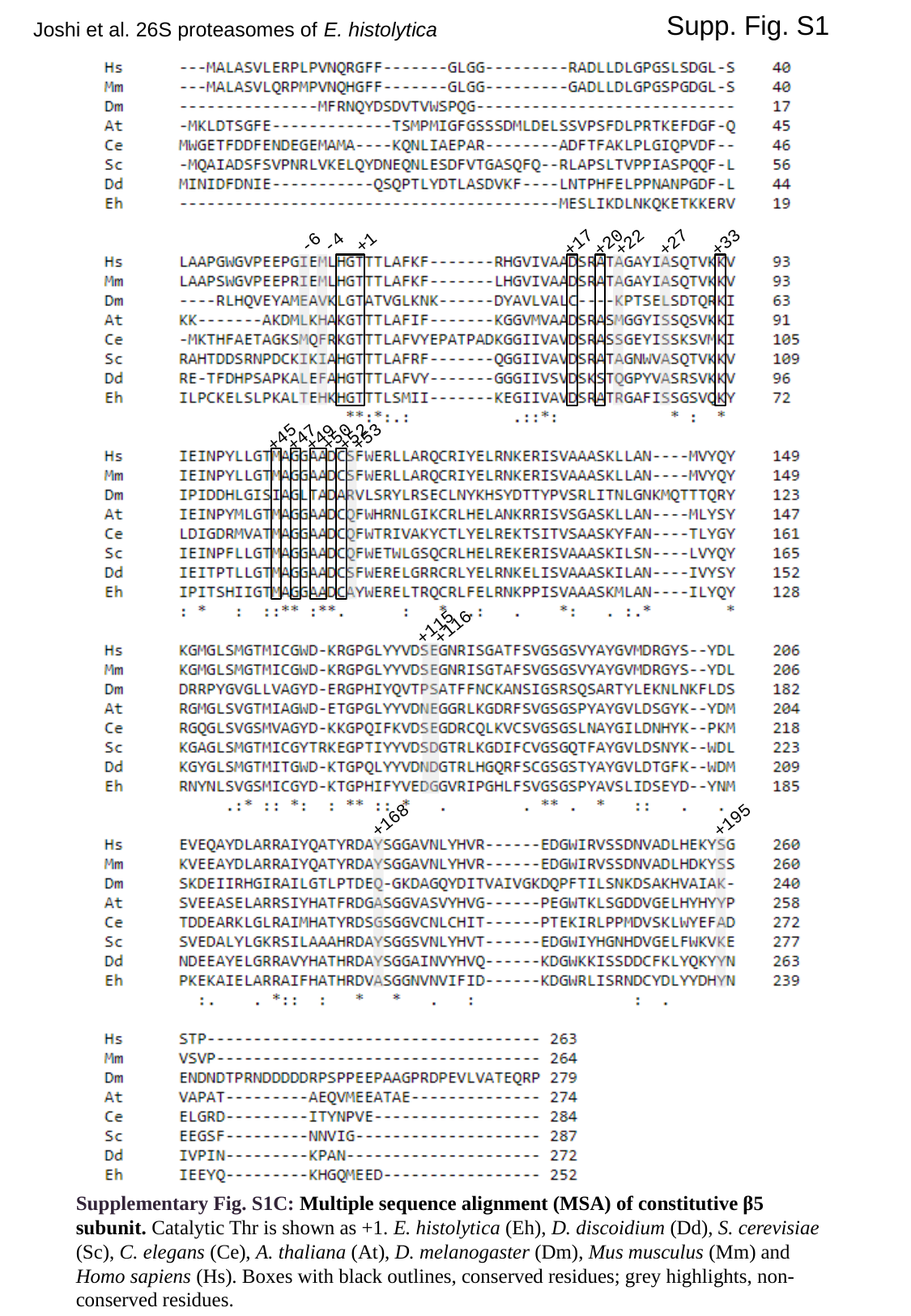

Supp. Fig. S1
Joshi et al. 26S proteasomes of E. histolytica
-6
-4
+1
+17
+20
+22
+27
+33
+45
+47
+49
+50
+52
+53
+115
+116
+168
+195
Supplementary Fig. S1C: Multiple sequence alignment (MSA) of constitutive β5 subunit. Catalytic Thr is shown as +1. E. histolytica (Eh), D. discoidium (Dd), S. cerevisiae (Sc), C. elegans (Ce), A. thaliana (At), D. melanogaster (Dm), Mus musculus (Mm) and Homo sapiens (Hs). Boxes with black outlines, conserved residues; grey highlights, non-conserved residues.

## Slide 4
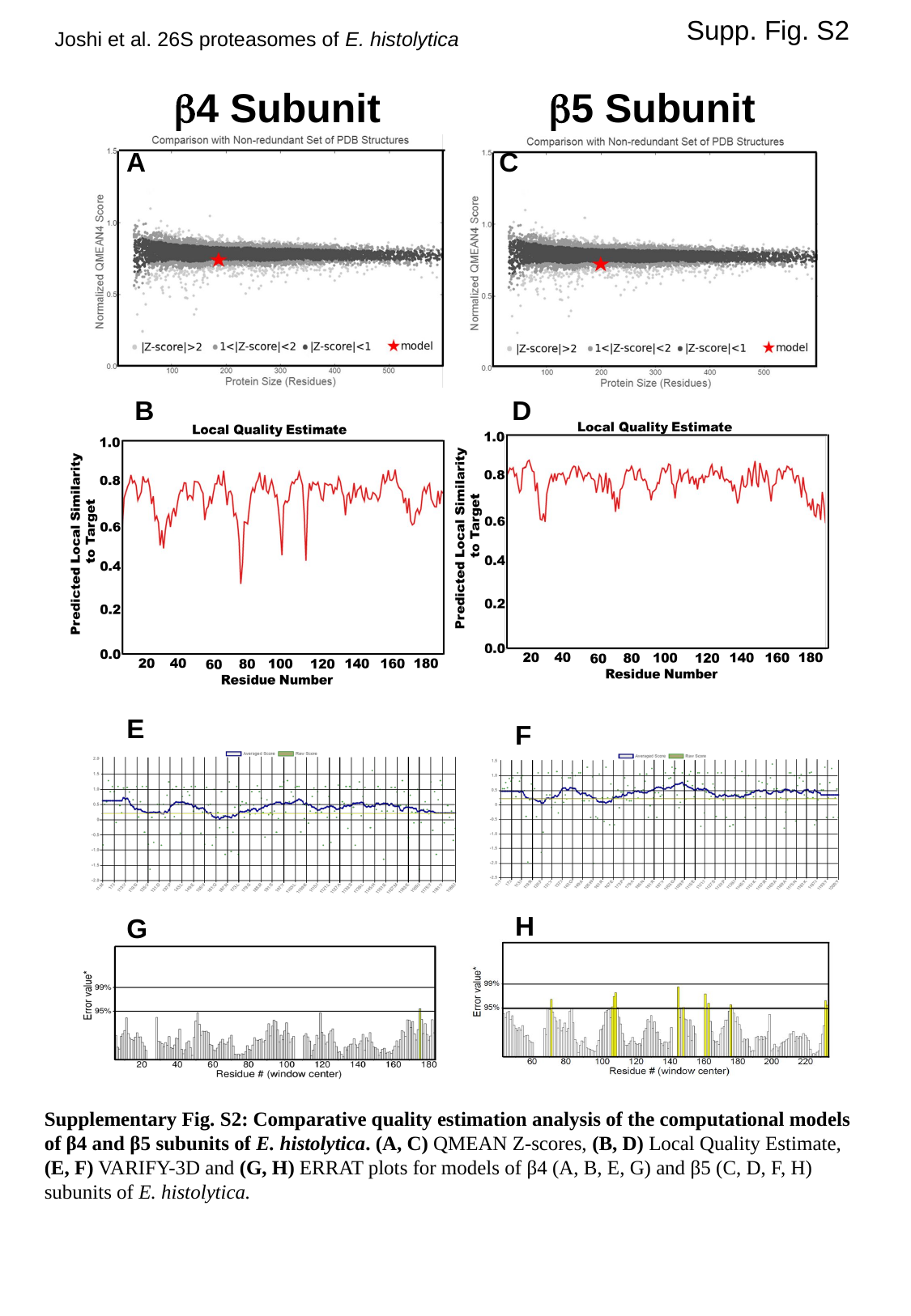

Supp. Fig. S2
Joshi et al. 26S proteasomes of E. histolytica
b4 Subunit
b5 Subunit
A
C
B
D
E
F
H
G
Supplementary Fig. S2: Comparative quality estimation analysis of the computational models of β4 and β5 subunits of E. histolytica. (A, C) QMEAN Z-scores, (B, D) Local Quality Estimate, (E, F) VARIFY-3D and (G, H) ERRAT plots for models of β4 (A, B, E, G) and β5 (C, D, F, H) subunits of E. histolytica.

## Slide 5
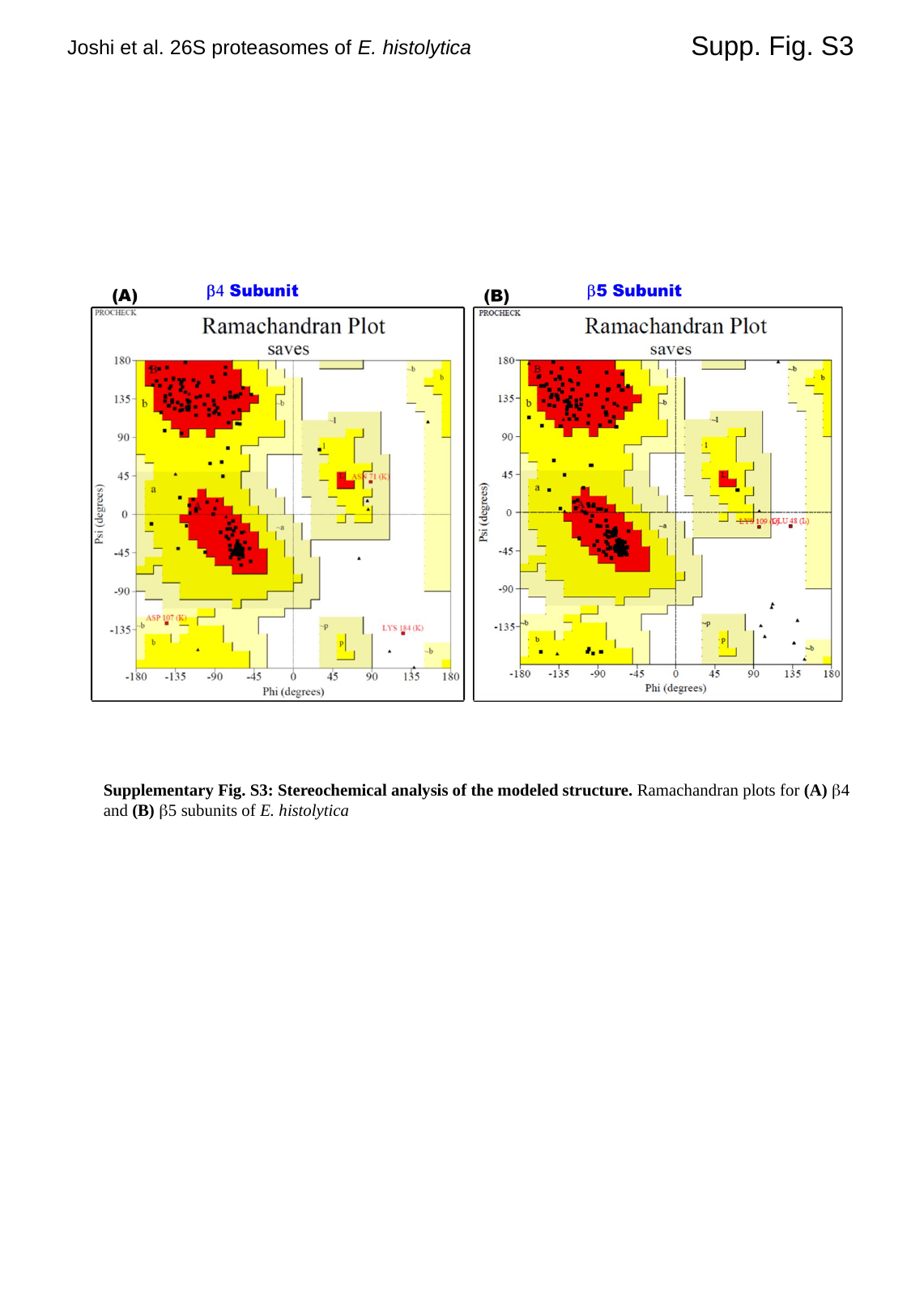

Supp. Fig. S3
Joshi et al. 26S proteasomes of E. histolytica
Supplementary Fig. S3: Stereochemical analysis of the modeled structure. Ramachandran plots for (A) b4 and (B) b5 subunits of E. histolytica

## Slide 6
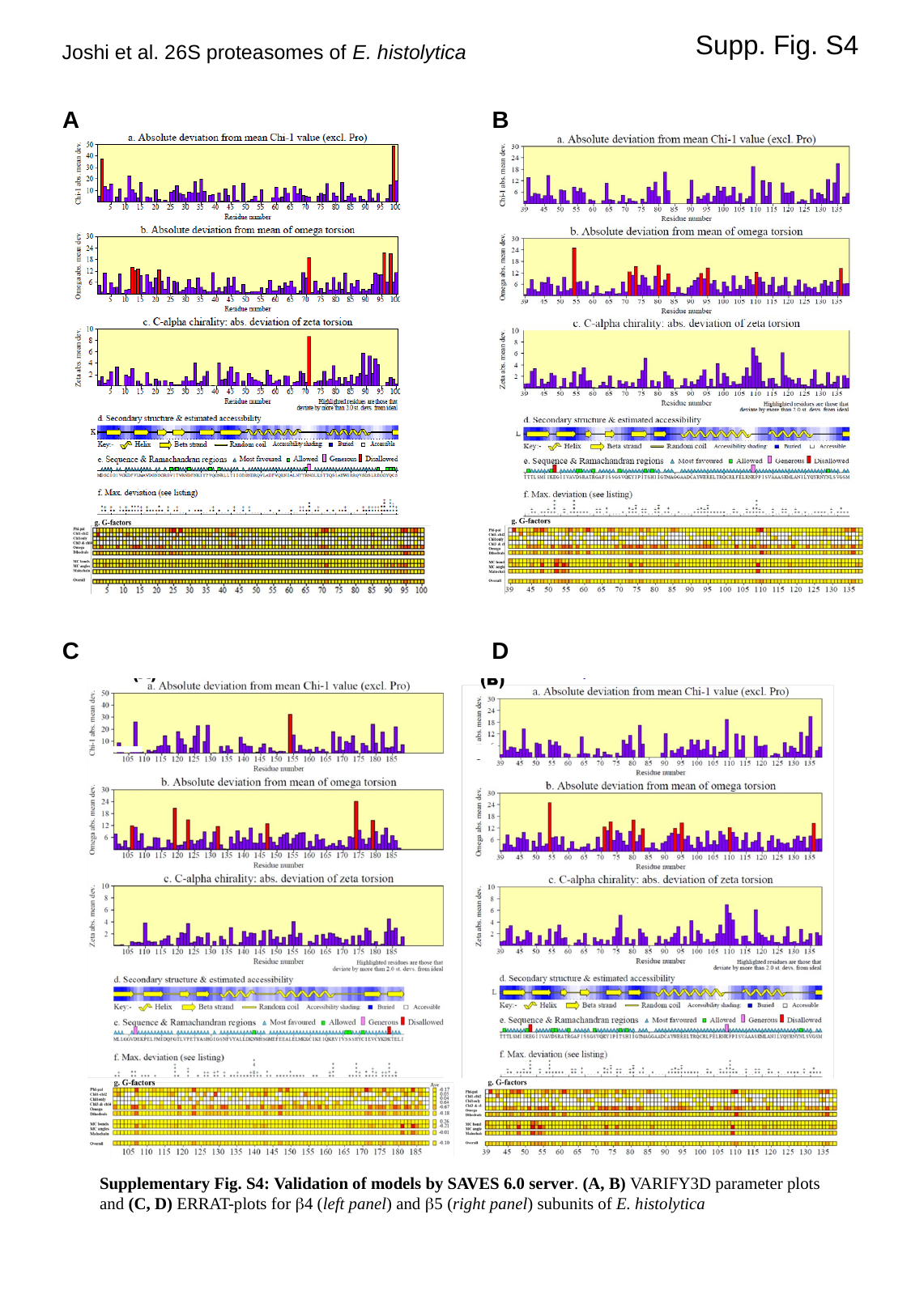

Supp. Fig. S4
Joshi et al. 26S proteasomes of E. histolytica
A
B
C
D
Supplementary Fig. S4: Validation of models by SAVES 6.0 server. (A, B) VARIFY3D parameter plots and (C, D) ERRAT-plots for b4 (left panel) and b5 (right panel) subunits of E. histolytica

## Slide 7
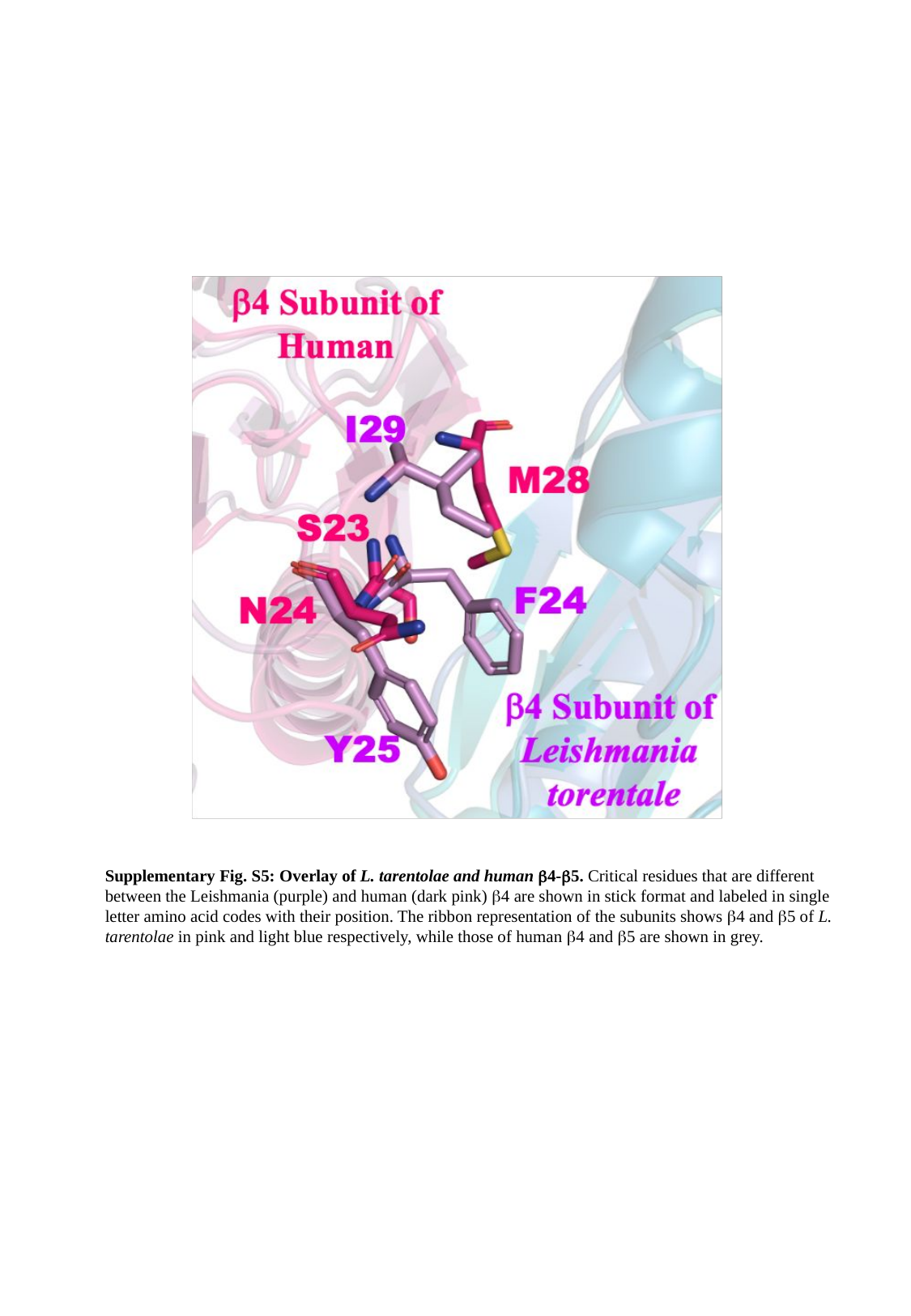

Supplementary Fig. S5: Overlay of L. tarentolae and human b4-b5. Critical residues that are different between the Leishmania (purple) and human (dark pink) b4 are shown in stick format and labeled in single letter amino acid codes with their position. The ribbon representation of the subunits shows b4 and b5 of L. tarentolae in pink and light blue respectively, while those of human b4 and b5 are shown in grey.
